# Bearing fault diagnosis based on Kepler algorithm and attention mechanism

**DOI:** 10.1371/journal.pone.0331128

**Published:** 2025-09-04

**Authors:** Yu Jie Guang, Xiao Shun Gen, Song Meng Meng, Yu Wen Hui, Fang Yan, Ying He Jie

**Affiliations:** 1 School of Mechanical and Electrical Engineering, ningde normal university, Ningde City, Fujian Province, China; 2 School of Information Engineering, ningde normal university, Ningde City, Fujian Province, China; 3 School of Mechanical and Electrical and Engineering, Ningde Normal University, Ningde City, Fujian Province, China; 4 Shenzhen Jinxin Technology Co., Ltd, Runheng Dingfeng High tech Industrial Park, Shenzhen, Guangdong Province, China; 5 Fu’an Emergency Management Bureau, Ningde Emergency Management Bureau, Ningde City, Fujian Province, China; Ningbo University, CHINA

## Abstract

As a crucial component in rotating machinery, bearings are prone to varying degrees of damage in practical application scenarios. Therefore, studying the fault diagnosis of bearings is of great significance. This article proposes the Kepler algorithm to optimize the weights of neural networks and improve the diagnostic accuracy of the model. At the same time, combined with attention mechanisms, the model will focus on useful information, ignore useless information, and efficiently extract key features. Finally, using third-party bearing data and inputting it into the fault diagnosis model, it was verified that Kepler algorithm and attention mechanism can improve the diagnostic accuracy. Meanwhile, the algorithm proposed in this paper was compared with other algorithms to verify its feasibility and superiority.

## 1. Introduction

As a crucial component in rotating machinery, bearings have the advantages of compact structure, light weight, strong load-bearing capacity, and low cost. In practical application scenarios, bearings are subjected to complex dynamic heavy load forces and are susceptible to varying degrees of damage, resulting in different types of failure [1]. Meanwhile, the majority of mechanical equipment failures are directly caused by bearing failures, accounting for 40% −50% [2,3]. Therefore, studying the fault diagnosis of bearings is of great significance. In recent years, this issue has received widespread attention from scholars both domestically and internationally.

When diagnosing bearing faults, traditional fault diagnosis methods and artificial intelligence fault diagnosis methods can be used. The traditional fault diagnosis method is to use sensors to collect data on bearing operation, manually extract features from the operation data, and finally use the extracted features to train a machine learning model that can distinguish different types of fault features [4–6]. At present, traditional fault diagnosis mainly includes K-nearest neighbor algorithm, support vector machine, and artificial neural network. Due to its simple principle, K-Nearest Neighbor (KNN) algorithm is often used in early bearing fault diagnosis.Song et al. [7] proposed a fault detection method based on standardized KNN, which characterizes the distance between data and its neighbors through a standardized distance,this method requires a large amount of computation and is inefficient because for each text to be classified, its distance to all known samples must be calculated. In order to reduce computational complexity and improve efficiency, the support vector machine (SVM) method was adopted in later fault diagnosis.Chen et al. [8,9] proposed an early fault diagnosis method based on orthogonal neighborhood preservation embedding and Adaboost SVM algorithm. The orthogonal neighborhood preservation embedding method is used to eliminate redundant information in the original multi domain feature set, and then SVM is improved into Adaboost SVM for early fault diagnosis. However, this method relies heavily on the operator’s professional knowledge. In order to reduce the dependence of the model on the operator’s professional knowledge,Rex et al. [10,11]proposed a hybrid method for extracting and classifying gear faults by integrating Hu invariant moments and artificial neural networks. However, this method is inefficient and labor-intensive. In short, traditional fault diagnosis methods have obvious drawbacks, as they require a large amount of manpower and specialized knowledge in the corresponding field, resulting in low accuracy. In addition, there may be errors in the diagnosis process due to subjective factors.

Meanwhile, artificial intelligence can also be applied in the field of bearing fault diagnosis, achieving excellent results in the field of fault diagnosis [12–15]. Among them, neural networks, as a branch of artificial intelligence, are widely used in fault detection of bearings [16–19]. Jin Zhihao et al. [20]used neural networks for bearing fault detection. This method takes the original time-domain vibration signal as input, converts the data form using Welch power spectrum while suppressing high-intensity noise, and then trains the convolutional neural network with the obtained power spectrum. Finally, the trained model is used for bearing fault diagnosis. However, this method requires preprocessing of irrelevant data, and the diagnosis process is relatively complicated. In order to reduce the tedious data preprocessing process, Gao Feng et al. [21]established a neural network-based fault diagnosis model to achieve adaptive extraction of fault features. Although this method can reduce the preprocessing process, the neural network structure used is relatively simple, which makes the diagnosis process more complicated and requires multiple training of the model. In order to reduce the number of training models, Chang Miao et al. [22] proposed a fault diagnosis algorithm based on an improved neural network.Improving the structure of the neural network model by adding a new convolutional layer before the fully connected layer improves the structure of the neural network. However, this method does not optimize the weights of the neural network, and when dealing with numerous data with small differences, the neural network cannot concentrate and efficiently capture feature data.

In order to solve the weight optimization problem in the neural network structure and improve the attention of the neural network, this paper uses Kepler algorithm to optimize the weights in the neural network, and applies attention mechanism to enhance the attention of the neural network. The optimized neural network model is applied to bearing fault diagnosis, which improves the efficiency and accuracy of bearing fault diagnosis.

## 2. Basic knowledge

### 2.1. Kepler algorithm

In dealing with complex problems and optimization fields, some traditional optimization methods, such as gradient descent and genetic algorithms, have achieved good results in many problems. However, when dealing with complex multimodal optimization problems and highly nonlinear problems, they still expose slow convergence speed and local optima. Therefore, new optimization algorithms have emerged to better solve complex problems in modern science and engineering. The inspiration for Kepler Optimization Algorithm (KOA) comes from Kepler’s laws of planetary motion, which apply the laws of planetary motion to the algorithm and design a new type of swarm intelligence optimization algorithm. Its emergence provides engineers with a novel and unique way to solve optimization problems, especially in solving complex numerical optimization and machine learning parameter tuning, demonstrating good performance. The advantages of KOA algorithm:

(1) Strong global search capability: The KOA algorithm can comprehensively explore the search space by simulating the orbital motion of planets, avoiding getting stuck in local optimal solutions
(2) Fast convergence speed: KOA algorithm can find the optimal solution in a short time, improving diagnostic efficiency
(3) Good parameter optimization effect: By optimizing the hyperparameters of neural network, the KOA algorithm significantly improves the accuracy and efficiency of bearing fault diagnosis.

Traditional metaheuristic algorithms such as genetic algorithm (GA), particle swarm optimization algorithm (PSO), and ant colony algorithm (ACO) have shown excellent performance in solving complex optimization problems, but they have some shortcomings:

(1) Easy to get stuck in local optima: These algorithms may converge too early during the search process and cannot find the global optimum
(2) Slow convergence speed: Traditional metaheuristic algorithms have a slow convergence speed when dealing with large-scale problems, which affects diagnostic efficiency

It is precisely because the KOA algorithm has the above advantages that the KOA algorithm superior over traditional metaheuristics for this specific bearing diagnosis task. In this article, we apply the Kepler algorithm to optimize the weights of neural network. Optimizing the weights of neural network through Kepler algorithm can converge to the optimal value more quickly, thereby improving the training speed and prediction accuracy of the model.

The basic principle of KOA [23] is derived from Kepler’s laws of planetary motion. The three laws of KOA summarize the laws of planetary motion around the sun. KOA has created a mathematical model based on position and velocity by simulating the trajectory of planetary motion and the interaction of universal gravity [24]. The two main aspects of algorithms include:

(1) Gravitational attraction: Planets move around their orbits due to the universal gravitational force of the Sun. KOA adjusts their search direction by calculating the gravitational interaction between particles (representative solutions), enhancing their global search capability.
(2) Orbital characteristics: Different orbital features (such as ellipses, parabolas, etc.) are used to simulate different search states, making the optimization process diverse and flexible.

The basic principles of KOA algorithm [25,26] are described in detail as follows:

#### 2.1.1. Definition of gravity.

**Fig 1:**
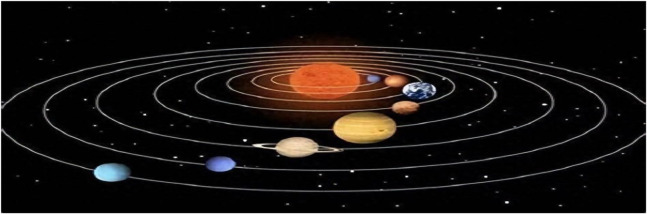
Planets orbiting the Sun.


As the largest celestial body in the solar system, the sun maintains the orbits of planets in elliptical orbits through universal gravity, as shown in Fig 1. The orbital velocity of a planet is inversely proportional to its distance from the sun: the closer the distance, the higher the velocity. These dynamics can be explained by the law of universal gravitation, which describes that the gravitational force between objects is proportional to their mass and inversely proportional to the square of their distance. The expression of universal gravitation is shown in formulas (1) and (2).

$$Fg_{i}(t)=e_{i}\times \mu (t)\times \frac{\overset{―}{M_{S}}\times \overset{―}{m_{i}}}{{\overset{―}{R_{i}}}^{2}+\varepsilon }+r_{1}$$ (1)

$$R_{i}(t)={\left\|X_{s}(t)-X_{i}(t)\right\|}_{2}=\sqrt{\sum_{j=1}^{d}{\left(X_{sj}(t)-X_{ij}(t)\right)}^{2}}$$ (2)

Among them, μ is the gravitational constant; $e_{i}$ is the eccentricity of a planet’s orbit,it is a random value between 0 and 1; $\overset{―}{R_{i}}$ representing the Euclidean distance between the sun and planets; $\overset{―}{M_{s}}$ and $\overset{―}{m_{i}}$ represent the standardized values of $M_{s}$ and $m_{i}$; ε is a minimum value; The value of $r_{1}$ is between 0 and 1, which is a randomly generated value that provides more variation for the gravity value during the optimization process.

#### 2.1.2. Calculate the speed of an object.

When a planet approaches the sun, its velocity increases due to the strong gravitational pull of the sun; When a planet moves away from the sun, its speed decreases due to the weakening of gravity, and this dynamic behavior can be modeled through equations [27]. This model consists of two parts: adjusting the distance between solutions to adjust the velocity of planets close to the sun, in order to enhance search diversity; Reduce these distances to decrease the speed of planets away from the sun, improve the problem of insufficient solution diversity, and enhance solution diversity. The velocity expression of a planet can be represented by formulas (3) – (14).


$$V_{i}(t)=\left\{ \begin{array}{c} \ell \times \left(2r_{4}\vec{X_{i}}-\vec{X_{b}}\right)+\ddot{\ell }\times \left(\vec{X_{a}}-\vec{X_{b}}\right)+\left(1-R_{i-norm}(t)\right)\times \Gamma \\ \times \vec{U_{1}}\times \vec{r_{5}}\times \left(\vec{X_{i,up}}-\vec{X_{i,low}}\right),ifR_{i-norm}(t)\leq 0.5\\ r_{4}\times \zeta \times \left(\vec{X_{a}}-\vec{X_{i}}\right)+\left(1-R_{i-norm}(t)\right)\times \Gamma \times U_{2}\times \vec{r_{5}}\times \left(r_{3}\vec{X_{i,low}}\right),else \end{array}\right.$$ (3)

$$\ell =\vec{U}\times M\times \varsigma$$ (4)

$$\varsigma ={\left[\mu \left(t\right)\times \left(M_{s}+m_{i}\right)\left|\frac{2}{R_{i}\left(t\right)+\varepsilon }-\frac{1}{a_{i\left(t\right)}+\varepsilon }\right|\right]}^{\frac{1}{2}}$$ (5)

$$M=\left(r_{3}\times \left(1-r_{4}\right)+r_{4}\right)$$ (6)

$$\vec{U}=\left\{ \begin{array}{c} 0\;\vec{r_{5}}\leq \vec{r_{6}}\\ 1\;Else \end{array}\right.$$ (7)

$$\Gamma =\left\{ \begin{array}{c} 1,ifr_{4}\leq 0.5\\ -1,Else \end{array}\right.$$ (8)

$$\ddot{\ell }=\left(1-\vec{U}\right)\times \vec{M}\times \varsigma$$ (9)

$$\vec{M}=\left(r_{3}\times \left(1-\vec{r_{5}}\right)+\vec{r_{5}}\right)$$ (10)

$$\vec{U_{1}}=\left\{ \begin{array}{c} 0\;\vec{r_{5}}\leq r_{4}\\ 1\;Else \end{array}\right.$$ (11)

$$U_{2}=\left\{ \begin{array}{c} 0\;r_{3}\leq r_{4}\\ 1\;Else \end{array}\right.$$ (12)

$$a_{i}\left(t\right)=r_{3}\times {\left[{T_{i}}^{2}\times \frac{\mu \left(t\right)\times \left(M_{s}+m_{i}\right)}{4\pi^{2}}\right]}^{\frac{1}{3}}$$ (13)

$$T_{i}=\left|r\right|,i=1,.....N;$$ (14)

Among them, $V_{i}(t)$ represents the velocity of the object i in time t, $X_{i}$ represents the planet i, $r_{3}$ and $r_{4}$ is a randomly generated value in the interval [0,1]. $\vec{r_{5}}$ and $\vec{r_{6}}$ are two vectors, random values between 0 and 1; $\vec{X_{a}}$ and $\vec{X_{b}}$ represent solutions randomly selected from the group; $M_{s}$ and $m_{i}$ represents the mass of $X_{s}$ and $X_{i}$; μ(t) is the constant of universal gravitation; ε is a small value used to prevent zero division error; $R_{i}(t)$ representing the distance between the optimal solution $X_{s}$ and the object $X_{i}$ at time t; $a_{i}$ representing the semi major axis of the elliptical orbit of the object i at time t; $T_{i}$ representing the orbital period of the object i; r is a value randomly generated based on a normal distribution.

#### 2.1.3. Jumping out of local optima.

KOA draws inspiration from the natural behavior of planets in the solar system rotating around the sun and introduces a marker Γ to change the search direction, which can effectively escape from local optimal areas and enhance the comprehensive exploration capability of the entire space [28].

#### 2.1.4. Update target location.

**Fig 2:**
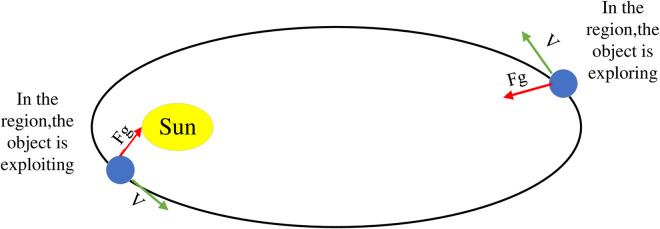
Exploration and development process of planets.


As shown in Fig 2, KOA divides the simulation of the natural motion of celestial bodies around the sun in elliptical orbits into two stages: exploration and development. In the exploration phase, KOA explores areas far from the sun to find new solutions. During the development phase, KOA focuses on utilizing known solutions close to the sun. The update of the target position can be represented by formula (15):

$$\vec{X_{i}}(t+1)=\vec{X_{i}}\left(t\right)+\Gamma \times \vec{V_{i}}(t)+\left(F_{g_{i}}\left(t\right)+\left|r\right|\right)\times \vec{U}\times \left(\vec{X_{S}}\left(t\right)-\vec{X_{i}}\left(t\right)\right)$$ (15)

Among them, $\vec{X_{i}}\left(t+1\right)$ is the new position of the object i at time t+1; $V_{i}\left(t\right)$ representing the speed required for an object i to reach a new location; $X_{s}\left(t\right)$ representing the best position of the sun discovered so far; Γ used as a flag to change search direction.

#### 2.1.5. Update distance from the sun.

KOA optimizes exploration and development by simulating the natural changes in distance between the sun and planets, and adjusts its operating mode based on the values of adjustment parameters. The distance between the planet and the sun can be updated using formulas (16) and (17).


$$\begin{array}{c} \vec{X_{i}}\left(t+1\right)=\vec{X_{i}}\left(t\right)\times \vec{U_{1}}+\left(1-\vec{U_{1}}\right)\times \\ \left(\frac{\vec{X_{i}}\left(t\right)+\vec{X_{s}}+\vec{X_{a}}\left(t\right)}{3.0}+h\times \left(\frac{\vec{X_{i}}\left(t\right)+\vec{X_{s}}+\vec{X_{a}}\left(t\right)}{3.0}-\vec{X_{b}}\left(t\right)\right)\right) \end{array}$$ (16)

$$h=\frac{1}{e^{\eta r}}$$ (17)

Among them, h is an adaptive factor used to control the distance between the sun and the current planet at time t, η is a linearly decreasing factor from 1 to −2.

**Fig 3:**
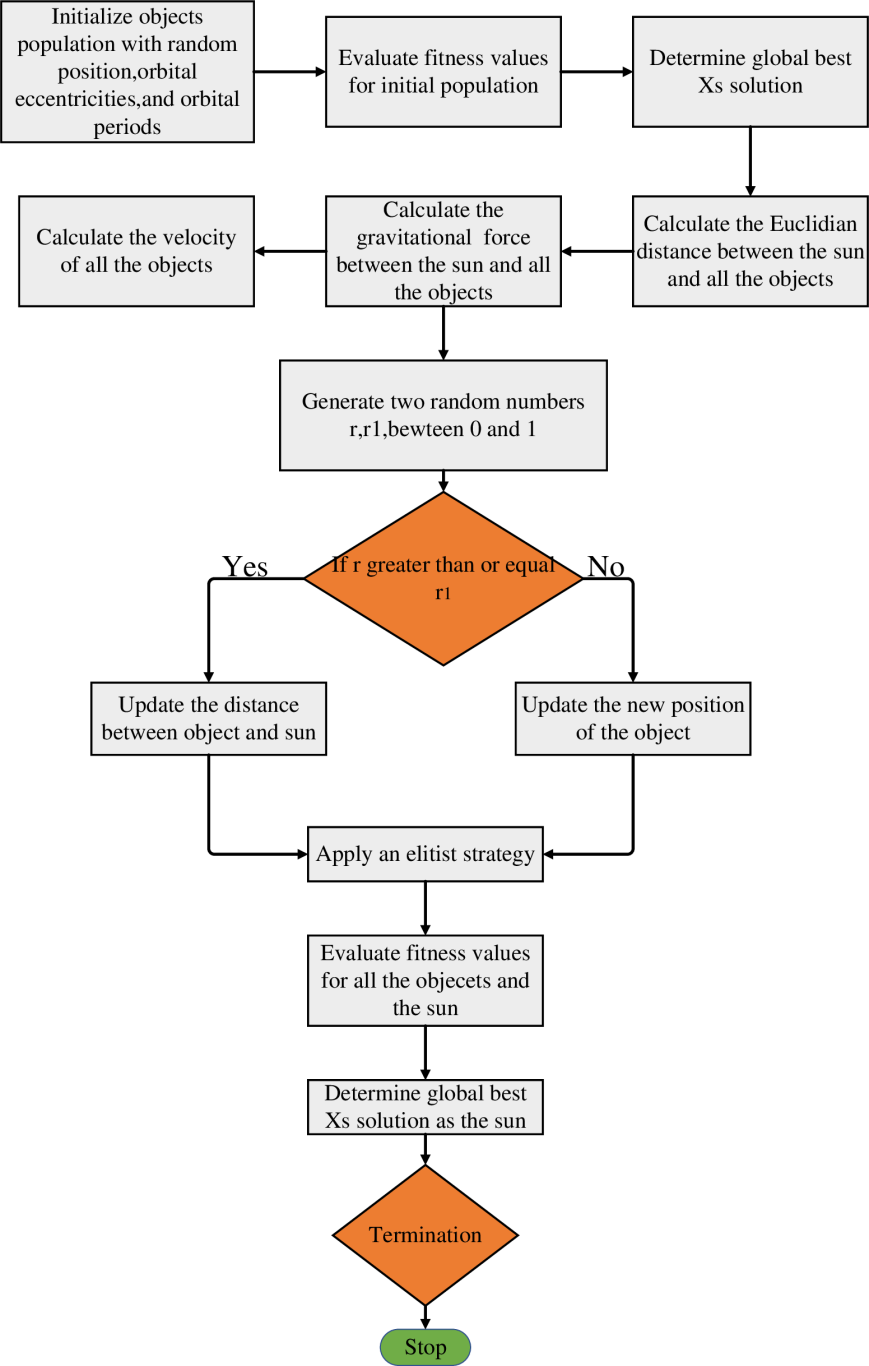
Kepler algorithm flowchart.

In summary, the entire calculation process of Kepler algorithm is shown in Fig 3:

### 2.2 Neural networks

Neural networks [29]mimic the data processing process of human neurons to process data. Neural networks generally include convolution operations, which have the characteristics of sparse connections, parameter sharing, and translation invariance. Neural networks are mainly composed of several fully connected layers,convolutional layers, and pooling layers, which will be introduced in this article.

#### 2.2.1 Fully Connected Layer.

**Fig 4:**
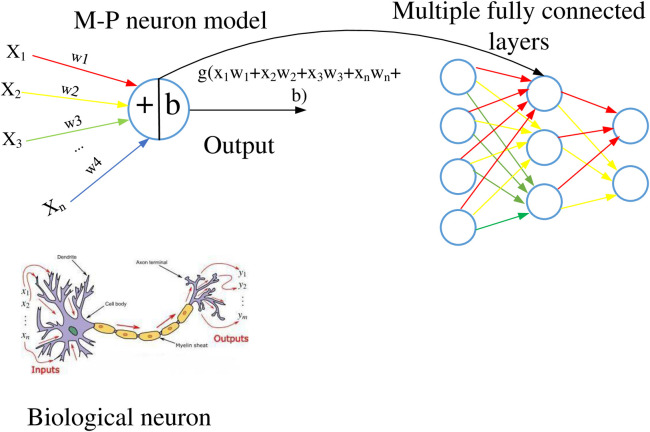
M-P neuron model and fully connected layer structure.

The fully connected layer [30] is composed of multiple M-P neurons, as shown on the right side of Fig 4, with a chain like structure between layers. Usually, the number of neurons in the next layer (layer width) is chosen to be less than or equal to the number of neurons in the previous layer, in order to compress the high-dimensional data of the convolutional neural network into lower dimensional data (feature extraction) for subsequent classifier classification.

M-P neuron is a mathematical model abstracted from biological neurons, and the output expression of a single neuron is shown in equation (18).


$$y=g\left(x_{1}w_{1}+x_{2}w_{2}+...+x_{n}w_{n}+b\right)=g\left(\sum_{i=1}^{n}w_{i}x_{i}+b\right)$$ (18)

Among them, $X_{i}$ represents the input component, b is the bias, $W_{i}$ is the weight on the corresponding arc on the right side of the neuron model. The input data passes through the arc and is multiplied by the weight, then input to the neuron for summation, and finally activated by the hidden unit (activation function) to obtain the output of the neuron. From the calculation process, it can be seen that weights will affect the final result, so optimizing their weights is very necessary.

#### 2.2.2. Convolutional layer.

The convolutional layer uses convolution operations between the input matrix and the weight matrix instead of matrix multiplication [31]. Convolution operation is a mathematical operation on two time-varying functions, where and are the real variable functions of, known as kernel functions. Due to the fact that the data processed by computers is discrete, the discrete expressions for convolution operations are shown in formulas (19) and (20).


(x*w)(T)=∫x(t)w(T−t)dt (19)

$$\left(x*w\right)\left(T\right)=\sum_{t=0}^{N}x\left(t\right)w\left(T-t\right)$$ (20)

**Fig 5:**
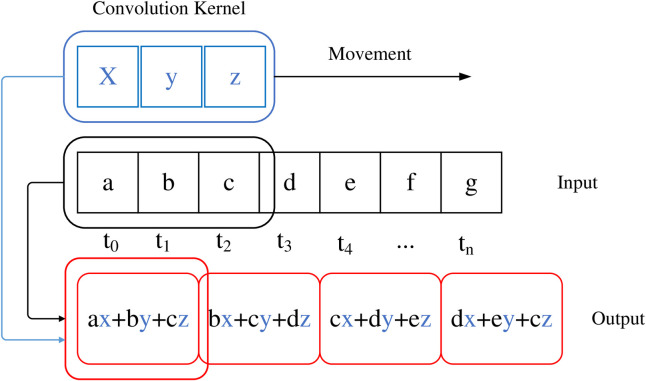
Process of One Dimensional Convolution Operation.

In the formula, T represents the current convolution time point, t represents the time scale of the time series, and N is the length of the time series, w is the convolution kernel and also a type of weight.Different weights will greatly affect the final calculation result. From the calculation process, it can be seen that weights will affect the final result, so optimizing their weights is very necessary.This article focuses on one-dimensional time series, and the calculation process can refer to Fig 5.

#### 2.2.3. Pooling layer.

The pooling layer replaces the input with local statistics [32], and commonly used statistics in the pooling process include maximum mean and norm. The formula for the pooling process can be represented by formula (21).


$${p_{j}}^{\left(l\right)}=\underset{js\leq i\leq js+W-1}{\max}\left\{{x_{i}}^{\left(l-1\right)}\right\}j=0,1...,\left\lceil \frac{N-W}{S}\right\rceil +1$$ (21)

**Fig 6:**
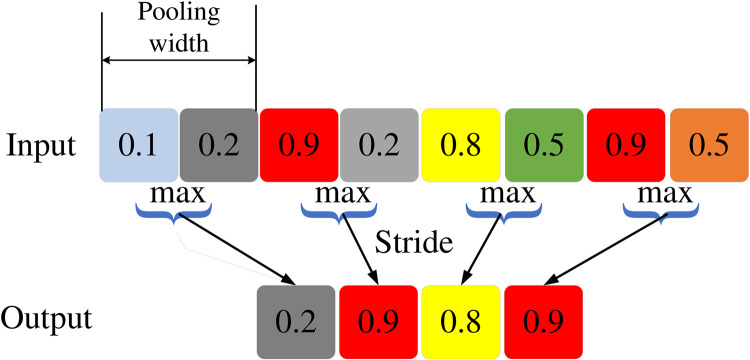
One dimensional max pooling process.

Among them, ${p_{j}}^{\left(l\right)}$ represents the j th output component of the pooling layer l. ${x_{i}}^{\left(l-1\right)}$ represents the i th input component of the layer l−1_,_ is the pooling width, W is the pooling stride, S is the pooling stride, N is the sum of the lengths of the input sequence, and ⌈⌉ represents rounding up. The maximum statistical value for the pooling process shown in Fig 6 is selected, with a pooling width of 2 and a pooling stride of 2. From the calculation process of the pooling layer, it can be seen that the pooling layer needs to select the maximum value in the data. If the data size of a long time series is relatively close during the data processing, it will be difficult for the pooling layer to select suitable data during the operation process, and it will also take a lot of time to perform the operation. To address this issue, attention mechanisms can be introduced to ignore redundant irrelevant data and focus attention on useful feature data.

### 2.3. Long short term memory network(LSTM)

There are many types of neural networks, and long short-term memory networks are one of them. Long Short Term Memory [33](LSTM) is a special type of neural network, which is a time recurrent neural network mainly used for processing and modeling sequence data, such as text, speech, time series, etc. Due to the fact that the bearing fault data used in this article is time series data, an LSTM network is employed. Due to the presence of fully connected layers and convolutional layers in the LSTM network structure, weights are used for mathematical operations in each layer, which can affect the final calculation results. Therefore, optimizing the weights of LSTM is crucial.

### 2.4. Attention mechanism

**Fig 7:**
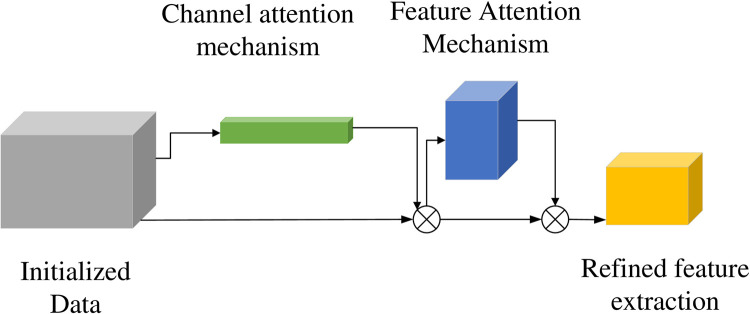
Attention mechanism model.

Due to the inability of the pooling layer in LSTM networks to handle massive amounts of duplicate data, this paper introduces an attention mechanism to address this issue. The attention mechanism is essentially a resource allocation mechanism that changes the way resources are allocated based on the importance of the attention target, tilting resources more towards the attention object [34]. Adding attention mechanism to object detection tasks can enhance the representation ability of the model, reduce the interference of invalid targets, improve the detection effect of attention targets, and improve the accuracy of model detection. According to the characteristics of attention mechanism, it can solve the pain points of LSTM network processing duplicate data. It can make LSTM network more focused and efficient, and can match LSTM network very well.The model is shown in Fig 7 below:

## 3. Fault diagnosis model based on Kepler algorithm and attention mechanism

**Fig 8:**
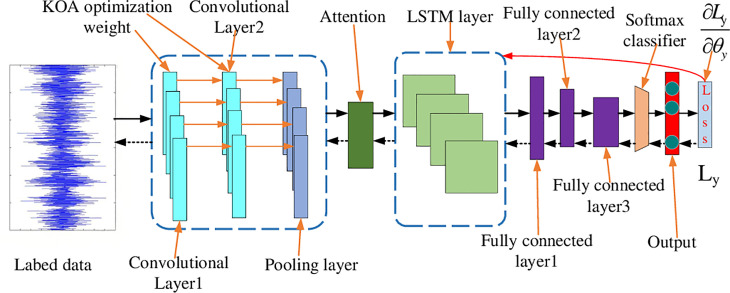
Fault diagnosis model based on Kepler algorithm and attention mechanism.

Based on the above description, it can be seen that neural networks have two major pain points. In view of this, this paper proposes a new diagnostic model based on LSTM network, optimizes its weights using Kepler algorithm, and combines attention mechanism to improve its ability to process duplicate data, solving its two major pain points. This model extracts features from a large amount of one-dimensional vibration data through convolution, and then reduces the dimensionality through pooling. The pooled data uses attention mechanism to fully extract its features, and then outputs the final diagnostic result. Meanwhile, throughout the entire diagnostic process, the weights of the neural network will be optimized using the Kepler algorithm. The fault diagnosis model used in this article has a structure as shown in Fig 8:

## 4. Experimental analysis

### 4.1. Experimental data

**Fig 9:**
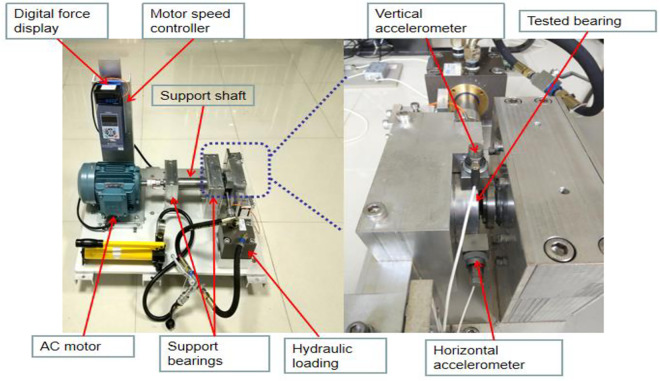
Experimental collection platform.

The most objective way to evaluate the superiority of the algorithm proposed in this article is to use a third-party standard database [35]and compare the prediction results of this algorithm with current mainstream algorithms. This article selects rolling bearing data from Xi’an Jiaotong University, and the fault data of the bearings is obtained through artificial damage setting and accelerated experiments [36]. The experimental collection platform is shown in Fig 9. The experimental equipment includes an AC motor, a motor speed controller, a motor speed controller, two support bearings (heavy-duty load roller bearings), a hydraulic loading system, etc. During the acceleration testing experiment, three different operating conditions were set, and five bearings were tested under each operating condition. The operating conditions set are as follows:

(1) 2100 revolutions per minute (35 Hz) and 12 kilonewtons;
(2) 2250 revolutions per minute (37.5 Hz) and 11 kilonewtons;
(3) 2400 revolutions per minute (40 Hz) and 10 kilonewtons.

**Fig 10:**
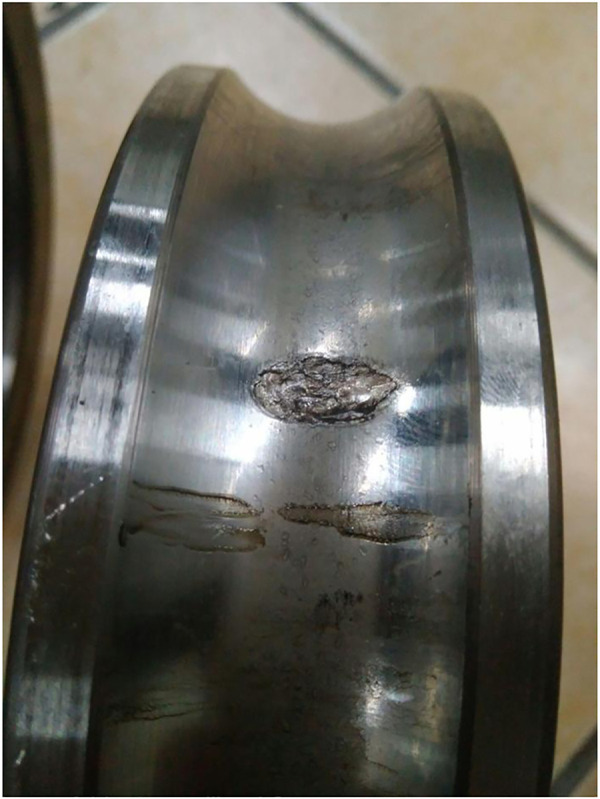
Damage to Inner.

**Fig 11:**
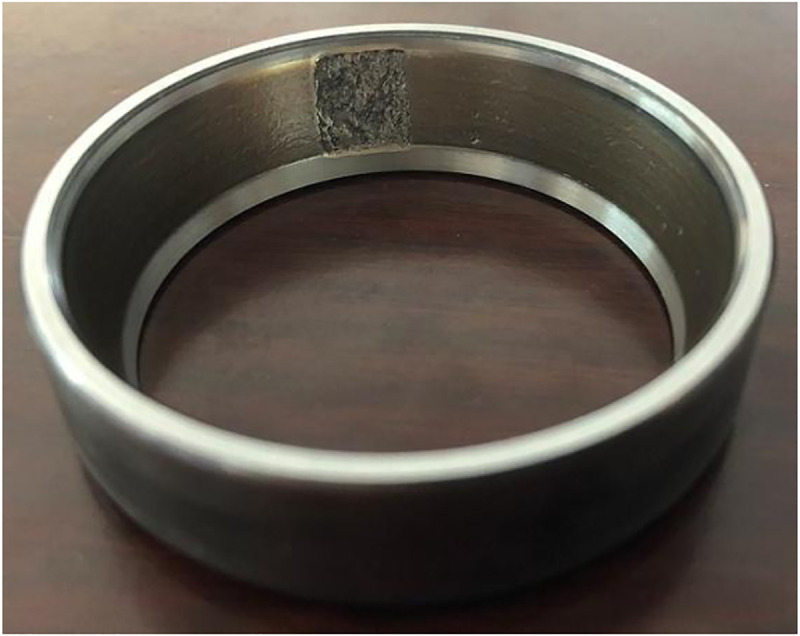
Damage to Ring of Bearing the outer ring of the bearing.

**Fig 12:**
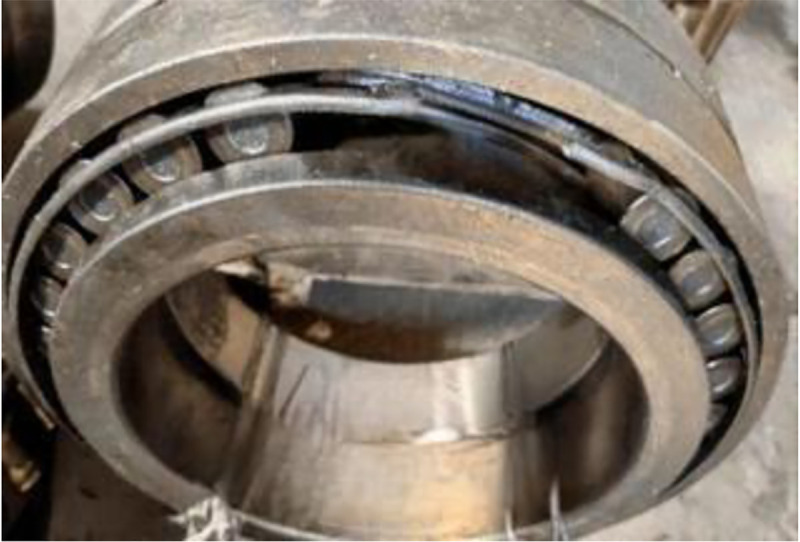
Damaged Bearing Retainer.

The final collected experimental results are as follows, with a total of 9 experimental data states. When diagnosing bearing faults, undamaged normal bearing data was added, with a total of 10 data states. The normal bearing data was named category 10, and the naming of the bearing data is detailed in Table 1 below. The damage to the bearings after the experiment is shown in Figs 10–12.

**Table 1:** Grouping of Bearing Data.

Operating condition	Bearing dataset	Number of files	Bearing lifetime	Fault element	Fault category
Condition 1 (35 Hz/12kN)	Bearing 1_1	123	2h 3 min	Outer race	1
	Bearing 1_2	161	2h 41 min	Outer race	1
	Bearing 1_3	158	2h 38 min	Outer race	1
	Bearing 1_4	122	2h 2 min	Cage	2
	Bearing 1_5	52	52min	Inner race and Outer race	3
Condition 2 (37.5 Hz/11kN)	Bearing 2_1	491	8h 11 min	Inner race	4
	Bearing 2_2	161	2h 41 min	Outer race	5
	Bearing 2_3	533	8h 53 min	Cage	6
	Bearing 2_4	42	42min	Outer race	5
	Bearing 2_5	339	5h 39 min	Outer race	5
Condition 3 (40 Hz/10kN)	Bearing 3_1	2538	42h 18 min	Outer race	7
	Bearing 3_2	2496	41h 36 min	Inner race, ball,cage and Outer race	8
	Bearing 3_3	371	6h 11 min	Inner race	9
	Bearing 3_4	1515	25h 15 min	Inner race	9
	Bearing 3_5	114	1h 54 min	Outer race	7

### 4.2. Model parameter

#### 1. Data preprocessing.

All input features are normalized using [min max normalization] before training, and reduced to the [0,1] interval.

Samples with missing values have been removed to ensure data integrity.

All classification labels have been encoded with integers (such as category labels encoded as 1, 2, 3, etc.).

#### 2. Training/testing set partitioning.

The original dataset is randomly divided into a training set and a testing set, with a segmentation ratio of 80%/20%.

#### 3. Neural network training parameters.

Maximum number of iterations (Epochs): 500

Batch size: 32 samples per batch

This model adopts a double-layer one-dimensional convolution structure (convolution kernel size of 3x1, channel numbers of 32 and 64 respectively), integrates SE attention mechanism (channel compression ratio of 1/4), and is combined with single-layer LSTM (unit number of 6) and self attention module. The final classification output is achieved through fully connected layers and Softmax function. During training, the Adam optimizer is used with an initial learning rate of 0.01, which decays to 0.1 after every 100 rounds of training. The L2 regularization coefficient is 0.01, and the maximum training is 500 rounds. Before each round of training, the sample order will be randomly shuffled.

Input the fault data of the bearings into the model in this article, and the output structure size of each layer of the neural network is shown in Table 2 below:

**Table 2:** Neural Network Structure Parameters.

number	network layer	Convolutional kernel size	Number of convolution kernels	Output Size
1	input	/	/	238 × 1
2	Convolution 1	3 × 1	32	26 × 13 × 2 × 238
3	Convolution 2	3 × 1	64	24 × 1 × 64 × 238
4	Global average pooling	/	32	1 × 1 × 32 × 238
5	Attention layer	/	64	24 × 1 × 64 × 238
6	LSTM layer	/	/	6 × 238
7	Fully connected layer 1	/	16	1 × 1 × 16 × 238
8	Fully connected layer 2	/	64	1 × 1 × 64 × 238
9	Fully connected layer 3	/	1	10 × 238
10	Classification layer	/	/	10 × 238

### 4.3. Result analysis

To verify the effectiveness of the algorithm, a comparison was made between the model with Kepler algorithm and the model without Kepler algorithm to verify that Kepler algorithm can improve the accuracy of the model. At the same time, compare the models with and without attention mechanism to verify that the attention mechanism can extract important information features and improve the accuracy of the model.

**Fig 13:**
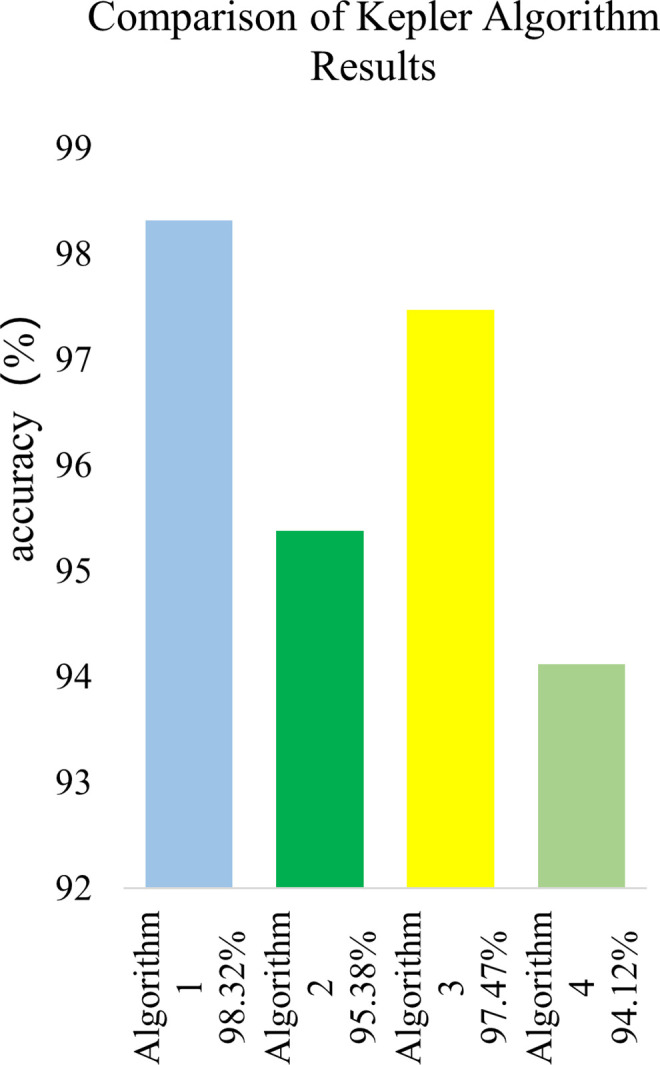
Comparison of Kepler algorithm results.

**Fig 14:**
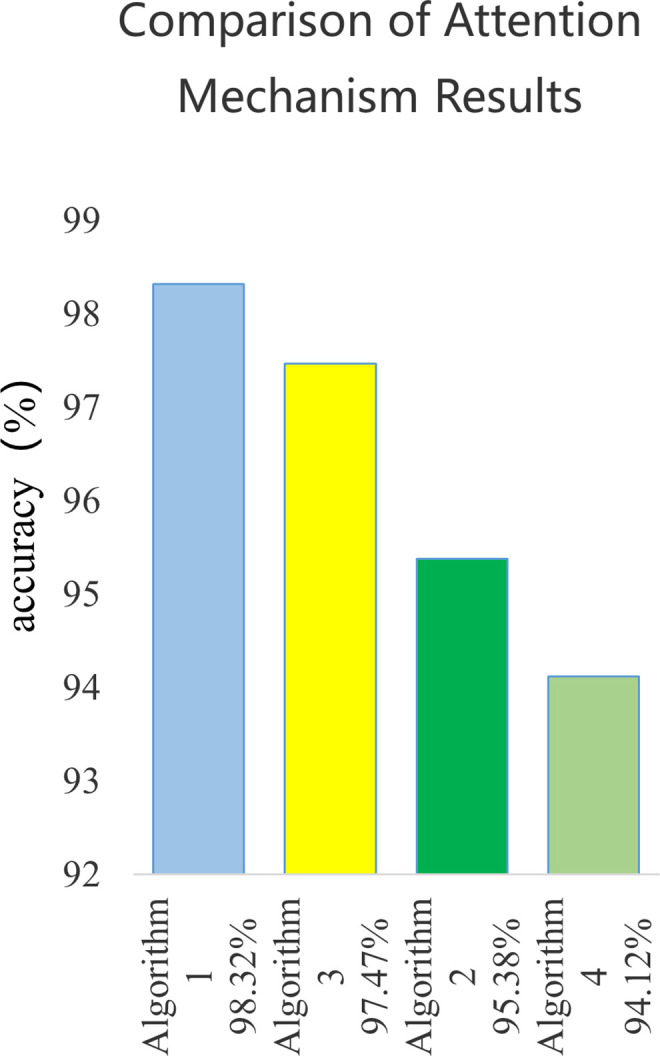
Comparison of Attention Mechanism Results.

This article uses four algorithms, namely KOA LSTM Attention (Algorithm 1), LSTM Attention (Algorithm 2), KOA LSTM (Algorithm 3), and LSTM (Algorithm 4), to predict fault classification results. The results are compared and summarized in Figs 13 and 14.

The accuracy of the four algorithms is 98.32%, 95.38%, 97.47%, and 94.12%, respectively. Algorithm 1 has a 2.94% higher accuracy than Algorithm 2, and Algorithm 3 has a 3.35% higher accuracy than Algorithm 4. The comparison of the two sets of results fully demonstrates that Kepler algorithm can optimize the weights of neural networks and improve the accuracy of the model. The accuracy of

Algorithm 1 is 0.85% higher than that of Algorithm 3, and the accuracy of Algorithm 2 is 1.26% higher than that of Algorithm 4. The comparison results of the two sets of data fully demonstrate that the attention mechanism can effectively process massive duplicate data, improve the model’s focus, and enhance the accuracy of the improved model.

**Fig 15:**
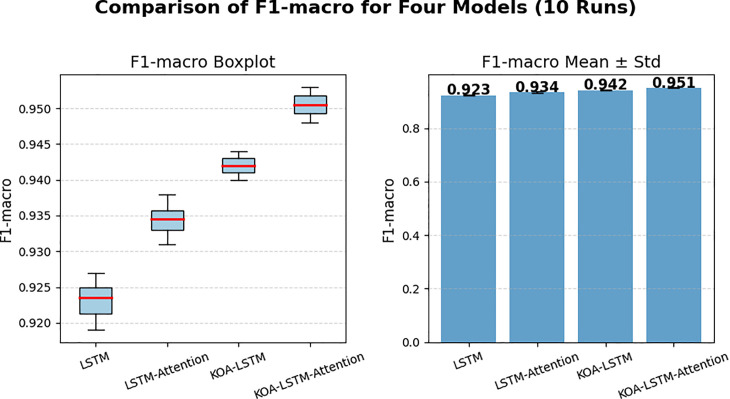
Comparison of F1-macro for four Models.

To further analyze the performance of each model, we calculated the F1 score of four algorithms in 10 independent experiments, and the results are shown in Table 3. And a box plot (F1 macro Boxplot) and a mean ± standard deviation bar chart (F1 macro Mean ± Std) were plotted in Fig 15. From the box plot, it can be seen that the F1 macro distribution of the KOA-LSTM Attention model is generally higher than other models, with smaller variance and more stable performance. The mean bar chart also shows that the model has the highest mean, and the difference is statistically significant.

**Table 3:** The results of multiple tests.

Repeat Counter	LSTM	LSTM- Attention	KOA- LSTM	KOA-LSTM-Attention
1	0.948	0.970	0.916	0.968
2	0.937	0.965	0.915	0.964
3	0.951	0.964	0.925	0.9770
4	0.939	0.971	0.930	0.9670
5	0.936	0.959	0.933	0.972
6	0.937	0.968	0.917	0.964
7	0.944	0.968	0.925	0.973
8	0.941	0.961	0.923	0.966
9	0.939	0.964	0.914	0.974
10	0.947	0.940	0.943	0.985
Average	0.942	0.963	0.924	0.971

**Fig 16:**
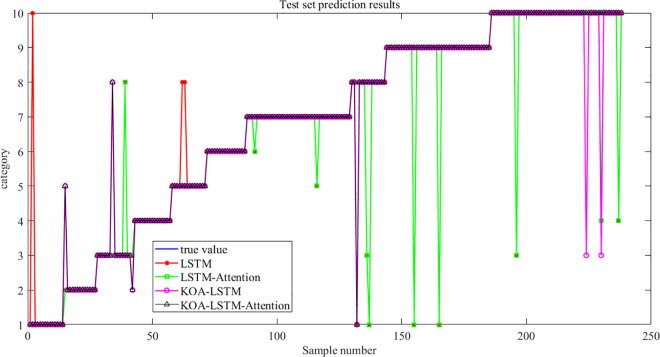
Test set prediction results of four algorithms.

Meanwhile, based on the prediction results of the test sets of the four algorithms shown in Fig 16, it can be concluded that the KOA-LSTM Attention algorithm has the fastest prediction results approaching the true values, which verifies the superiority of the proposed algorithm in this paper.

Meanwhile,independent experiments and performance comparisons were conducted for all three scenarios mentioned above, quantifying the respective contributions of KOA and attention mechanisms. The experimental results are shown in Table 4:

**Table 4:** Results of the Ablation Experiment.

Ablation Experiment	Precision	Recall	F1
KOA	0.933	0.957	0.942
Attention	0.959	0.968	0.963
KOA-Attention	0.915	0.935	0.924
KOA-LSTM-Attention	0.972	0.972	0.971

Due to the fact that the innovation of this article mainly focuses on the joint design of KOA mechanism and attention mechanism, the analysis of ablation experiments highlights the role of these two core parts in improving model performance and the comprehensive gain brought by their mutual cooperation.

**Fig 17:**
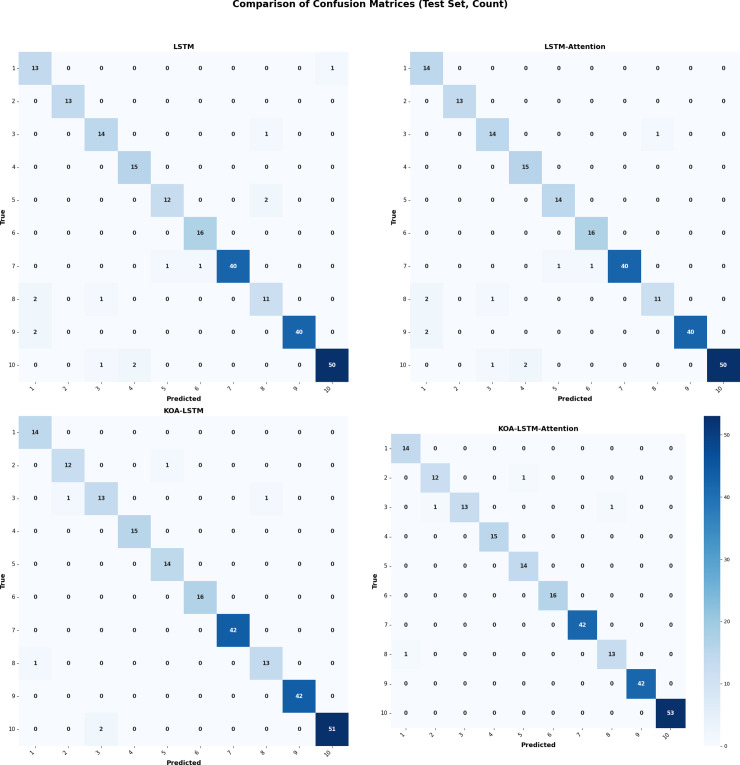
Confusion Matrix.

The confusion matrices of the four algorithms are shown in Fig 17

The precision, recall, and F1 score data for each category are shown in Table 5

**Table 5:** Evaluation parameter results.

Class	Precision	Recall	F1
1	0.933	1	0.966
2	0.923	0.923	0.923
3	1	0.867	0.929
4	1	1	1
5	0.933	1	0.966
6	1	1	1
7	1	1	1
8	0.929	0.929	0.929
9	1	1	1
10	1	1	1
Average	0.972	0.972	0.971

Through the F1 scores of each category, it can be seen that some small sample categories or easily mixed categories (such as categories 1 and 8) have lower scores, mainly due to uneven distribution of categories or similar features. With the improvement of the model, the accuracy and F1 of all categories have improved, especially KOA-LSTM Attention, which performs the best in each category and has the strongest overall performance. Combining the confusion matrix, further identify the main sources of misjudgment for certain categories and guide subsequent model optimization.

**Fig 18:**
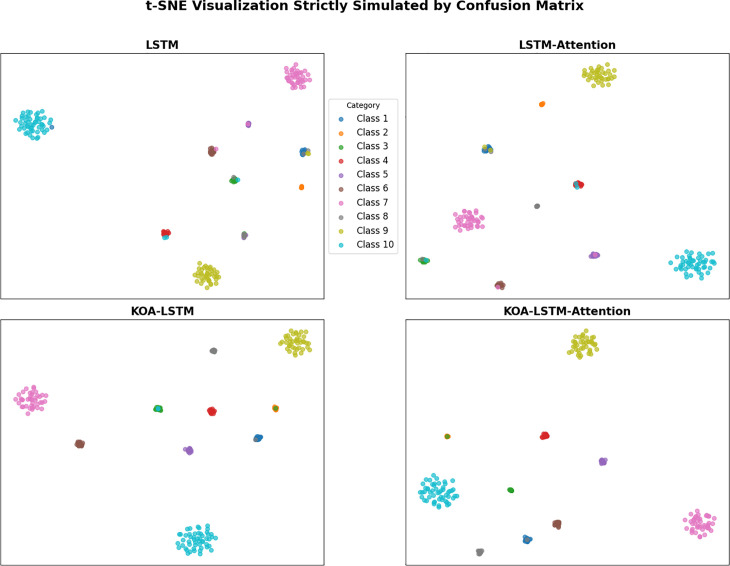
t-SNE Visualization strictly simulated by Confusion Matrix.

To further validate the feature extraction capability of the model, we have added t-SNE visualizations for each model’s feature extraction in Fig 18, which intuitively demonstrate the performance of each model in category differentiation from the perspective of dimensionality reduction distribution.

## 5. Conclusion

This article addresses the two major pain points of LSTM networks by applying Kepler algorithm and combined attention mechanism to solve their weight and attention problems. The optimized model is applied to the fault diagnosis of bearings, achieving efficient fault diagnosis. Finally, the accuracy of the four algorithms is compared to verify the feasibility and superiority of the algorithm proposed in this article.
